# CUDC‐907, a novel dual PI3K and HDAC inhibitor, in prostate cancer: Antitumour activity and molecular mechanism of action

**DOI:** 10.1111/jcmm.15281

**Published:** 2020-05-27

**Authors:** Cheng Hu, Hongyan Xia, Shanshan Bai, Jianlei Zhao, Holly Edwards, Xinyu Li, Yanrong Yang, Jing Lyu, Guan Wang, Yang Zhan, Yan Dong, Yubin Ge

**Affiliations:** ^1^ National Engineering Laboratory for AIDS Vaccine Key Laboratory for Molecular Enzymology and Engineering The Ministry of Education School of Life Sciences Jilin University Changchun China; ^2^ Department of Structural and Cellular Biology Tulane University School of Medicine Tulane Cancer Center New Orleans LA USA; ^3^ School of Nursing Jilin University Changchun China; ^4^ Department of Oncology Wayne State University School of Medicine Detroit MI USA; ^5^ Molecular Therapeutics Program Barbara Ann Karmanos Cancer Institute Wayne State University School of Medicine Detroit MI USA

**Keywords:** AKT, CUDC‐907, HDAC, PDX, PI3K, prostate cancer

## Abstract

Targeting the androgen receptor (AR) signalling pathway remains the main therapeutic option for advanced prostate cancer. However, resistance to AR‐targeting inhibitors represents a great challenge, highlighting the need for new therapies. Activation of the PI3K/AKT pathway and increased expression of histone deacetylases (HDACs) are common aberrations in prostate cancer, suggesting that inhibition of such targets may be a viable therapeutic strategy for this patient population. Previous reports demonstrated that combination of PI3K inhibitors (PI3KIs) with histone deacetylase inhibitors (HDACIs) resulted in synergistic antitumour activities against preclinical models of prostate cancer. In this study, we demonstrate that the novel dual PI3K and HDAC inhibitor CUDC‐907 has promising antitumour activity against prostate cancer cell lines in vitro and castration‐resistant LuCaP 35CR patient‐derived xenograft (PDX) mouse model in vivo. CUDC‐907‐induced apoptosis was partially dependent on Mcl‐1, Bcl‐xL, Bim and c‐Myc. Further, down‐regulation of Wee1, CHK1, RRM1 and RRM2 contributed to CUDC‐907‐induced DNA damage and apoptosis. In the LuCaP 35CR PDX model, treatment with CUDC‐907 resulted in significant inhibition of tumour growth. These findings support the clinical development of CUDC‐907 for the treatment of prostate cancer.

## 1 INTRODUCTION

Prostate cancer is the second most commonly diagnosed cancer and the second leading cause of death from cancer in men in the United States, with 174,650 new cases and 31,620 deaths estimated in 2019.^1^ The androgen receptor (AR) signalling pathway is critical for the growth and survival of prostate cancer cells, including those of lethal castration‐resistant prostate cancer (CRPC), demonstrating that AR is a valid therapeutic target for treating prostate cancer. Accordingly, androgen‐deprivation therapy has been the standard treatment for patients with metastatic disease, and the next‐generation AR‐targeting inhibitors, such as abiraterone and enzalutamide, have been the standard of care for patients with CRPC.^2^, ^3^, ^4^ However, resistance to these AR‐targeting therapies represents a great challenge for the treatment of these patients,^5^ highlighting the need for new therapies.

The PI3K pathway plays an important role in cancer cell proliferation, growth and survival ^6^ and is frequently activated in different types of cancer, including prostate cancer. Approximately 15% of primary prostate cancer and 50% of CRPC harbour alterations in this pathway, as determined by sequencing analysis.^7^, ^8^, ^9^ In addition, aberrant activation of the PI3K pathway is associated with poor prognosis, metastasis and resistance to therapy in prostate cancer patients.^10^, ^11^, ^12^ Although small molecule inhibitors targeting the PI3K pathway have demonstrated promising preclinical activity, clinical efficacy has been limited. These disappointing results are likely due to cross‐talk between the PI3K pathway and other survival pathways, suggesting that PI3K inhibitors must be used in combination with inhibitors targeting other survival pathways to ensure successful eradication of cancer cells.^13^, ^14^, ^15^, ^16^

Previous studies have shown that histone deacetylases (HDACs) are up‐regulated in prostate cancer and their expression levels correlate positively with Gleason scores and cell proliferation.^17^, ^18^ Preclinical studies have revealed that combination of PI3K inhibitors with HDAC inhibitors (HDACIs) show promising antitumour activity against solid tumours, including prostate cancer.^19^, ^20^, ^21^, ^22^ As a result, CUDC‐907, an orally available small molecule that dually inhibits HDAC (class I, II and IV, including HDAC1, 2, 3, 6, 10 and 11) and PI3K (class I α, β and δ) enzymes, was rationally designed and synthesized.^23^ CUDC‐907 has demonstrated substantial antitumour activity in multiple preclinical models of both solid tumours and haematological malignancies and clinical efficacy in relapsed/refractory lymphoma and multiple myeloma.^23^, ^24^, ^25^, ^26^, ^27^, ^28^, ^29^ Furthermore, CUDC‐907 is being tested in Phase I and II clinical trials for the treatment of multiple myeloma, lymphoma and advanced/relapsed solid tumours in adults and children (www.clinicaltrials.gov). CUDC‐907 was reported to be well tolerated at a paediatric recommended phase II dose administered orally, with mean T_max_ of 2.5 hours and T_1/2_ of 1.4 hours.^30^ In this study, we investigated CUDC‐907 in prostate cancer cells. We found that CUDC‐907 induces apoptosis in prostate cancer cells and that this is at least partially mediated by Mcl‐1, Bcl‐xL, Bim and c‐Myc. We also found that CUDC‐907 treatment induces DNA damage associated with down‐regulation of CHK1, Wee1 and the two subunits of ribonucleotide reductase (RR), RRM1 and RRM2. Further, our in vivo studies revealed that CUDC‐907 has potential for the treatment of prostate cancer.

## 2 MATERIALS AND METHODS

### 2.1 Drugs

CUDC‐907 (the chemical structure is shown in Figure S1), SCH772984 (an ERK‐selective inhibitor), LY2603618 (a CHK1‐selective inhibitor), MK‐1775 (a Wee1‐selective inhibitor), 10058‐F4 (a c‐Myc‐selective inhibitor) and Z‐VAD‐FMK (a pan‐caspase inhibitor) were purchased from Selleck Chemicals. Hydroxyurea (HU, a RR inhibitor) was purchased from Sigma‐Aldrich.

### 2.2 Cell culture

LNCaP, C4‐2, C4‐2B, LAPC‐4, PC‐3, DU145, 22Rv1 and HEK‐293T cells were purchased from the American Type Culture Collection (ATCC). LNCaP95 cells were kindly provided by Dr Alan Meeker at Johns Hopkins University. The cell lines were cultured in RPMI 1640 with 10% foetal bovine serum (FBS, Thermo Fisher Scientific) and 2 mmol/L L‐glutamine, plus 100 U/mL penicillin and 100 µg/mL streptomycin (except LNCaP95, which was cultured in RPMI 1640 with 10% charcoal‐stripped FBS), in a 37°C humidified atmosphere containing 5% CO2/95% air. Cell lines were regularly tested for the presence of mycoplasma utilizing the PCR method as described by Uphoff and Drexler.^31^ AR expression status is summarized in Table S1.

### 2.3 SRB assay

Viable prostate cancer cells were measured by using the SRB (Sulphorhodamine B, Sigma‐Aldrich) assay, as previously described.^32^ Briefly, the cells were treated with variable concentrations of CUDC‐907 for 72 hours. Cells were fixed with cold 10% (wt/vol) trichloroacetic acid for 3 hours and stained with SRB for 30 minutes, after which the excess dye was removed by washing with 1% (vol/vol) acetic acid. The cell‐bound SRB dye was dissolved in 10 mmol/L Tris base solution and plates were read at 515 nm using a microplate reader. IC_50_ values were calculated as drug concentrations necessary to inhibit 50% of cell growth compared to vehicle control‐treated cells using the GraphPad Prism 5.0 software. The IC_50_ values for the cell lines are presented as means of triplicates ± standard errors from at least three independent experiments.

### 2.4 Annexin V/PI staining and flow cytometry analysis

Prostate cancer cells were treated with the indicated drugs. Drug‐induced apoptosis was determined using the Annexin V‐Fluorescein Isothiocyanate (FITC)/Propidium Iodide (PI) Apoptosis Kit (Beckman Coulter) by following the manufacture's protocol. The experiments were repeated at least three times. Mean percentage of AnnexinV+/PI‐ (early apoptotic) and Annexin V+/PI+ (late apoptotic and/or dead) ± standard errors from triplicates of one representative experiment is shown.

### 2.5 Antibodies and western blot analysis

Anti‐H4, anti‐ac‐Tubulin, anti‐CDK1, anti‐p‐CDK1(Y15), anti‐CDK2, ‐p‐CDK2(Y15), anti‐RRM1, anti‐RRM2, and anti‐AKT (Abcam), anti‐p‐S6(S240/244), anti‐p‐CDC25C, anti‐Wee1, anti‐c‐Myc (Cell Signaling Technologies), anti‐β‐actin, anti‐PARP, anti‐Bcl‐2, anti‐Bax, anti‐Mcl‐1, anti‐ERK (Proteintech), anti‐p‐AKT (T308), anti‐p‐AKT (S473) (Affinity Biosciences), anti‐Bim, anti‐Bcl‐xL, anti‐Bak, anti‐p‐ERK(T202/Y204), anti‐CHK1 (Selleck Chemicals), anti‐ac‐H4 and anti‐γ‐H2AX (Millipore) antibodies were used for Western blot analyses.

Western blotting was performed as described previously.^33^ Briefly, whole cell lysates were prepared by sonication in 10 mmol/L Tris‐Cl, pH 7.0, containing 1% SDS, protease inhibitors and phosphatase inhibitors (Roche Diagnostics). The samples were separated by electrophoresis on SDS‐polyacrylamide gels and transferred onto polyvinylidene difluoride membranes (Thermo Fisher Scientific). After blocking in TBS buffer (150 mmol/L NaCl, 10 mmol/L Tris, pH 7.4) containing 5% fat‐free milk for 1 hour at room temperature, the blots were incubated with a primary antibody overnight at 4°C and then incubated with a fluorescent‐labelled secondary antibody for 1 hour at room temperature. Immunoreactive proteins were visualized using the Odyssey Infrared Imaging System (Li‐Cor). Western blots were repeated at least three times and one representative blot is shown.

### 2.6 Production of lentivirus particles and transduction of LNCaP cells

Non‐target control (NTC)‐, Bax‐, Bak‐ and Bim‐shRNA lentiviral vectors were purchased from Sigma‐Aldrich. RFP, Mcl‐1, Wee1, CHK1, RRM1, RRM2 and c‐Myc cDNA constructs were obtained from Dharmacon. Lentivirus production and transduction were carried out as previously described.^34^ Briefly, pMD‐VSV‐G, delta 8.2 and a lentiviral shRNA or cDNA construct were co‐transfected into HEK293T cells using polyethylenimine. Virus‐containing culture medium was harvested 48‐hour post‐transfection. LNCaP cells were infected by adding 1 mL of virus supernatant and 4 μg/mL of polybrene (Sigma‐Aldrich) and then cultured for an additional 48 hours prior to selection with 1 μg/mL puromycin for shRNA lentiviral vectors or 2 μg/mL blasticidin for overexpression lentiviral vectors, or treated with CUDC‐907 for certain experiments.

### 2.7 Alkaline comet assay

LNCaP and 22Rv1 cells were treated with CUDC‐907 for 18 hours and then subjected to alkaline comet assay, as previously described.^34^ Slides were stained with SYBR Gold (Thermo Fisher Scientific) and then imaged on an Olympus BX‐40 microscope equipped with a DP72 microscope camera and Olympus cellSens Dimension software (Olympus America Inc). Approximately 50 comets per gel were scored using CometScore (TriTek Corp). The median per cent DNA in the tail was calculated and graphed as mean ± standard error.

### 2.8 Quantification of gene expression by real‐time RT‐PCR

Total RNA was extracted using Total RNA Kit I (Omega Bio‐Tek). cDNAs were synthesized from 1 μg of total RNA by using the PrimeScript™ RT‐PCR Kit (Takara Bio USA) according to the manufacture's protocol. The real‐time RT‐PCR analysis was performed as described.^35^ *Bim* (Hs00708019_s1), Mcl‐1 (Hs01050896_m1), *Wee1* (Hs01119384_g1), *CHK1* (Hs00967506_m1) and *RRM2* (Hs00357247_g1) transcripts were quantitated using TaqMan probes (Life Technologies) and a LightCycler 480 real‐time PCR machine (Roche Diagnostics). *RRM1* transcripts were quantified using forward (5′‐ACTAAGCACCCTGACTATGCTATCC‐3′) and reverse (5′‐CTTCCATCACATCACTGAACACTTT‐3′) primers and SYBR green and the above‐mentioned real‐time PCR machine. *c‐Myc* transcripts were quantified using forward (5′‐GTGGTCTTCCCCTACCCTCT‐3′) and reverse (5′‐CGAGGAGAGCAGAGAATCCG‐3′) primers. Real‐time PCR results were expressed as means from three independent experiments and were normalized to that of the GAPDH transcript measured by either using a TaqMan probe (Hs02786624_g1) or forward (5′‐AGCCACATCGCTCAGACA‐3′) and reverse (5′‐GCCCAATACGACCAAATCC‐3′) primers and SYBR green. Fold changes were calculated using the comparative Ct method.^36^

### 2.9 LuCaP 35CR patient‐derived xenograft (PDX) mouse model

Male CB17 SCID mice were obtained from Charles River at 4‐6 weeks of age. After 1 week of adaptation, mice were castrated via a scrotal approach. On day 2 after castration, mice were inoculated subcutaneously with LuCaP 35CR tumour bits as described.^37^ When the tumours reached ~250 mm^3^, mice were randomly placed (5 mice/group) into the vehicle control or 100 mg/kg CUDC‐907 (3% ethanol (200 proof), 1% Tween‐80 (polyoxyethylene (20) sorbitan monooleate) and sterile water; all USP grade; v/v) group. Mice were treated daily via oral gavage for 19 days (total of 19 treatments). The tumour dimensions and bodyweights were measured every two days and every four days, respectively. Tumour volume was calculated as 0.524 × width^2^ × length.^38^ At the termination of the experiment (when one tumour in the vehicle control group reached 1000 mm^3^), mice were killed by CO2. All animal procedures were approved by the Tulane University Institutional Animal Care and Use Committee.

### 2.10 Statistical analysis

Statistical analyses were performed with GraphPad Prism 5.0. Error bars represent ± SEM. Statistical significance was determined with pair‐wise two‐sample *t* test (two‐tailed). The level of significance was set at *P* < .05.

## 3 RESULTS

### 3.1 CUDC‐907 treatment decreases viable cells and induces apoptosis in prostate cancer cell lines and shows antitumour activity in the LuCaP 35CR PDX model

**FIGURE 1:**
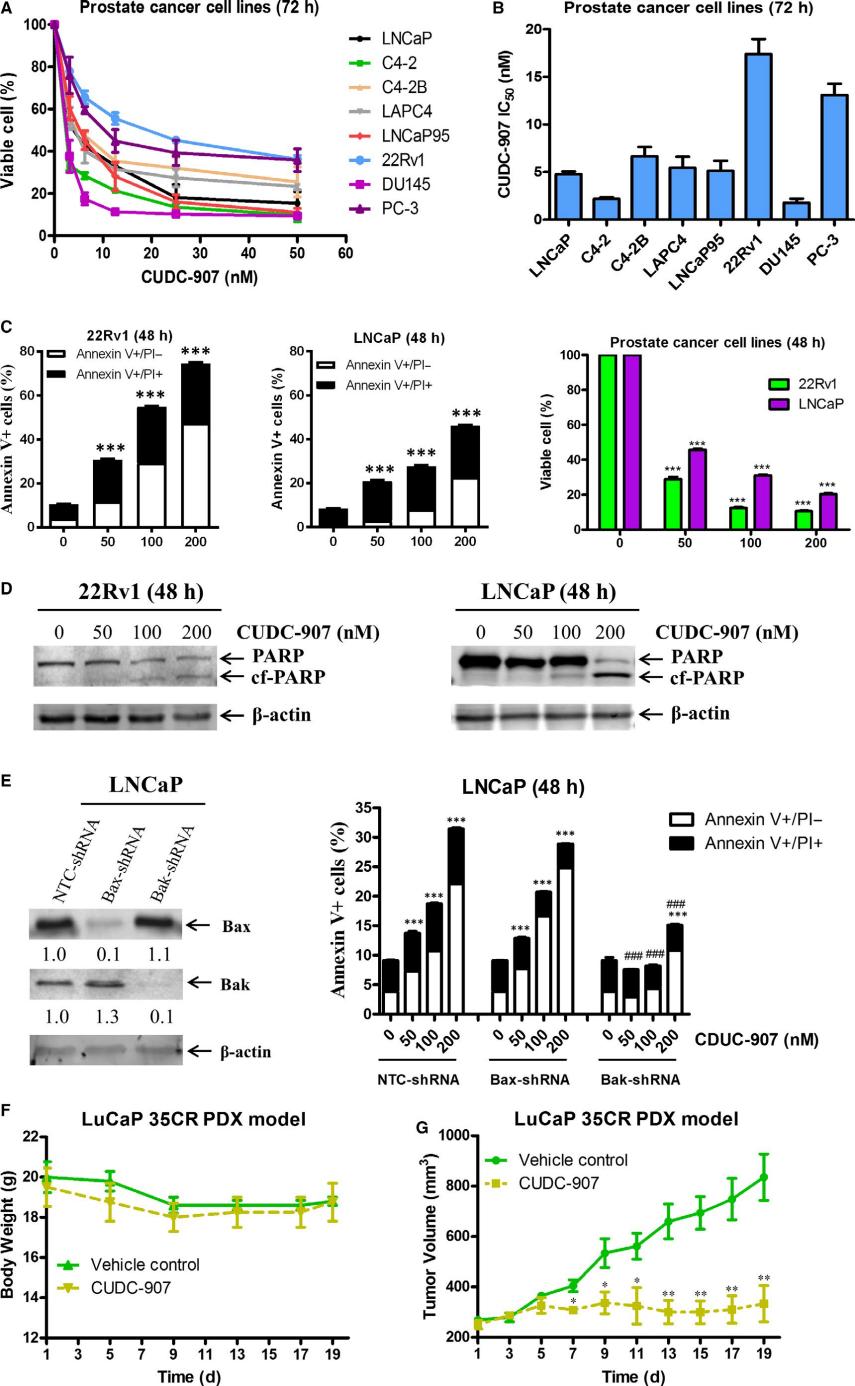
CUDC‐907 treatment decreases viable cells and induces apoptosis in prostate cancer cell lines, and shows promising antitumour activity in the LuCaP 35CR patient‐derived xenograft (PDX) model. A, Prostate cancer cell lines were treated with variable concentrations of CUDC‐907 in 96‐well plates for 72 h. The SRB assay was performed to determine viable cells. Viable cells in the vehicle control wells were set at 100%. Data are graphed as means of duplicates ± standard error from three independent experiments. B, IC50 values of CUDC‐907 from three independent experiments were calculated and graphed as means ± standard error. C, 22Rv1 and LNCaP cells were treated with variable concentrations of CUDC‐907 for 48 h and then subjected to Annexin V/PI staining and flow cytometry analysis. Data of apoptosis (left and middle panels) and viable cells (right panel) are presented as means of triplicates ± standard error from one representative experiment. ***indicates *P* < .001. D, 22Rv1 and LNCaP cells were treated as indicated in panel C. Whole cell lysates were subjected to Western blotting and probed with the indicated antibodies. cf‐PARP, cleaved PARP. E, LNCaP cells were infected with NTC‐, Bax‐ or Bak‐shRNA lentivirus for 5 h, then washed and cultured in fresh media for 19 h before treated with CUDC‐907 for 48 h. Knockdown of Bax or Bak was confirmed by Western blotting (left panel). The NTC‐shRNA, Bax‐shRNA and Bak‐shRNA knockdown cells treated with CUDC‐907 were subjected to Annexin V/PI staining and flow cytometry analysis (right panel). ***indicates *P* < .001 compared to the no drug treatment control, while ^###^indicates *P* < .001 compared to CUDC‐907‐treated NTC‐shRNA cells. F&G, LuCaP 35CR tumour bits were inoculated into castrated male nude mice. When the tumours reached ∼250 mm^3^, the mice were treated with 100 mg/kg CUDC‐907 via oral gavage for 19 d. Mean mouse bodyweights (panel F) and mean tumour volumes (panel G) are shown. *indicates *P* < .05, while **indicates *P* < .01 compared to the vehicle control group

To begin to test the antitumour activity of CUDC‐907 against prostate cancer cells, SRB assays were performed in 8 prostate cancer cell lines, including both androgen‐sensitive and castration‐resistant lines that are either AR‐positive or AR‐null. Cells were treated with increasing concentrations of CUDC‐907 for 72 hours. CUDC‐907 treatment resulted in reduction of viable cells in a concentration‐dependent manner in all the cell lines tested, with IC_50_s ranging from about 2 nmol/L to 17.4 nmol/L (Figure 1A,B). There is no clear difference in sensitivity of AR‐expressing versus AR‐null cells to CUDC‐907, indicating that the growth inhibition is unlikely dependent on AR. To determine if CUDC‐907 treatment causes death of prostate cancer cells, 22Rv1 and LNCaP cells were treated with 0‐200 nmol/L CUDC‐907 for 48 hours and then subjected to Annexin V/PI staining and flow cytometry analysis. CUDC‐907 treatment caused an increase in Annexin V positive cells and cleavage of PARP (Figure 1C left and middle panels and Figure 1D), demonstrating that CUDC‐907 treatment induced apoptosis in these cell lines. In addition, CUDC‐907 treatment also caused a decrease of viable cells, determined by flow cytometry analysis (Figure 1C right panel). To determine if CUDC‐907 induces apoptosis through the intrinsic apoptotic pathway, transient individual shRNA knockdown of Bak and Bax was performed in LNCaP cells. As shown in Figure 1E, shRNA knockdown of Bak, but not Bax, resulted in significant rescue of the cells from CUDC‐907 treatment, demonstrating that CUDC‐907‐induced apoptosis was at least partially through the intrinsic apoptotic pathway.

Next, we decided to determine the in vivo efficacy of CUDC‐907 in a castration‐resistant LuCaP 35CR PDX model. When the tumours reached ~250 mm^3^, mice were treated with either the vehicle control or 100 mg/kg CUDC‐907 daily via oral gavage. During the 19‐day period of treatment, CUDC‐907 treatment did not decrease bodyweight compared to the vehicle control group (Figure 1F), suggesting that the treatment was well tolerated. Starting on day 7 and continuing through the end of the treatment (when one tumour in the vehicle control group reached 1000 mm^3^), the difference in the average tumour size between the two groups was statistically significant (Figure 1G). At the end of CUDC‐907 treatment, the average tumour size in the vehicle control group was 835 mm^3^, while that in the CUDC‐907 group was 333 mm^3^, demonstrating a ∼60% inhibition of tumour growth by CUDC‐907 (Figure 1G). Taken together, the data shown in Figure 1 demonstrate that CUDC‐907 has promising antitumour activity against preclinical models of prostate cancer both in vitro and in vivo.

### 3.2 CUDC‐907 inhibits PI3K and HDACs but induces p‐ERK1/2 in prostate cancer cells

**FIGURE 2:**
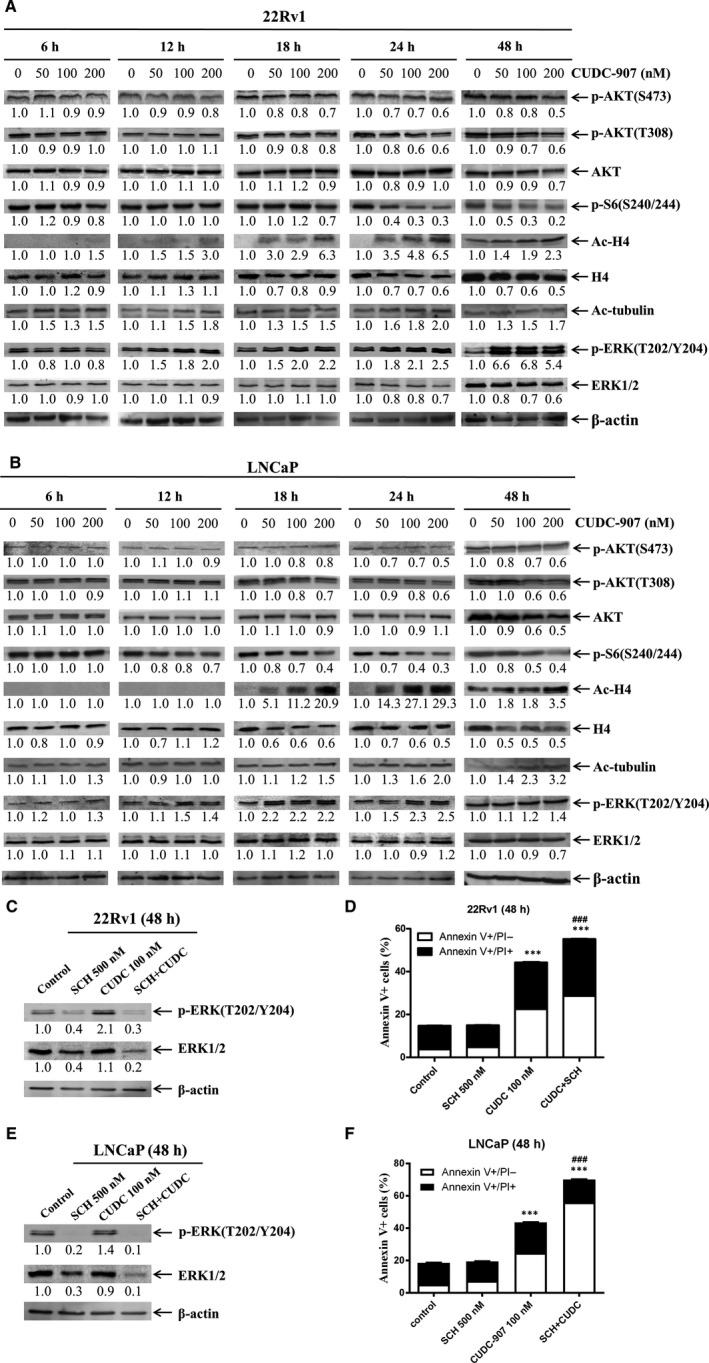
CUDC‐907 treatment inhibits PI3K and HDACs, but fails to inactivate ERK in prostate cancer cells. A&B, 22Rv1 (panel A) and LNCaP (panel B) cells were treated with variable concentrations of CUDC‐907 for 6‐48 h. Whole cell lysates were subjected to Western blotting and probed with the indicated antibodies. The fold changes for the densitometry measurements, normalized to β‐actin and then compared to no drug control, are shown below the corresponding blot. C‐F, 22Rv1 and LNCaP cells were treated with 100 nmol/L CUDC‐907 with or without 500 nmol/L ERK inhibitor SCH772984 for 48 h. Whole cell lysates of 22Rv1 (panel C) and LNCaP (panel E) cells were subjected to Western blotting. The CUDC‐907 treated 22Rv1 (panel D) and LNCaP (panel F) cells were subjected to Annexin V/PI staining and flow cytometry analyses. ***indicates *P* < .001 compared to no drug treatment control, while ^###^indicates *P* < .001 compared to individual drug treatment

To understand the molecular mechanism underlying the antitumour activity of CUDC‐907, we first determined the effects of CUDC‐907 on AKT/mTOR and ERK, pathways downstream of HDACs and PI3K, in 22Rv1 and LNCaP cells. CUDC‐907 treatment resulted in increased acetylation of histone H4 and alpha‐tubulin (deacetylated by HDAC6), confirming that CUDC‐907 possesses HDAC inhibitor activity (Figure 2A,B). CUDC‐907 treatment also decreased p‐AKT (both T308 and S473) and p‐S6 (downstream of AKT) levels as early as 18 hours, while total AKT levels remained largely unchanged in both cell lines (Figure 2A,B), demonstrating its PI3K inhibitor property. Surprisingly, CUDC‐907 treatment increased p‐ERK1/2 levels as early as 12 hours in both cell lines, but had no obvious impact on total ERK1/2 levels (Figure 2A,B). Given the role of ERK in cell proliferation and survival, this induction of p‐ERK1/2 may represent a potential mechanism of resistance to CUDC‐907. To test this possibility, the ERK1/2‐selective inhibitor SCH772984 was utilized to abrogate CUDC‐907‐induced up‐regulation of p‐ERK1/2 (Figure 2C,E), which significantly increased apoptosis induced by CUDC‐907 in both cell lines (Figure 2D,F). These results confirm that CUDC‐907 inhibits both HDACs and PI3K but fails to inactivate ERK1/2 in prostate cancer cells.

### 3.3 Mcl‐1, Bcl‐xL and Bim play important roles in CUDC‐907‐induced apoptosis

**FIGURE 3:**
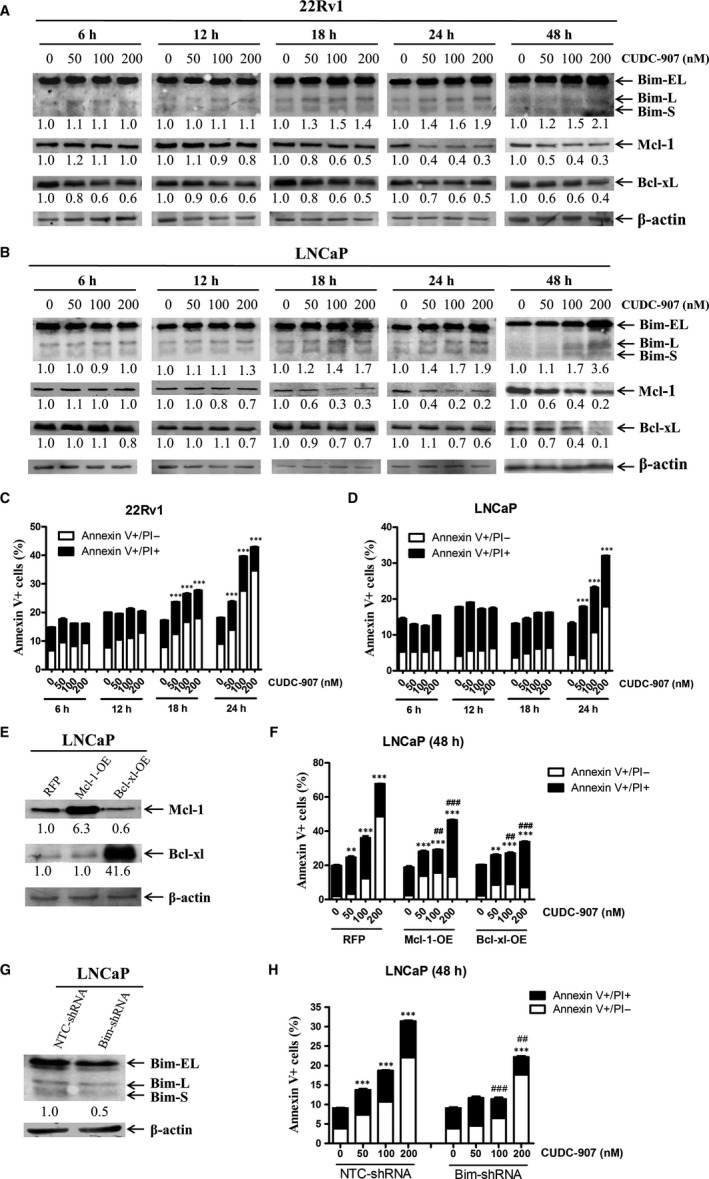
Mcl‐1, Bcl‐xL and Bim play important roles in CUDC‐907‐induced apoptosis in prostate cancer cells. A&B, 22Rv1 (panel A) and LNCaP (panel B) cells were treated with variable concentrations of CUDC‐907 for 6‐48 h. Whole cell lysates were subjected to Western blotting and probed with the indicated antibodies. The fold changes for the densitometry measurements, normalized to β‐actin and then compared to no drug control, are indicated below the corresponding blot. Bim S, L and EL indicate Bim short, long and extra‐long isoforms, respectively. C&D, 22Rv1 (panel C) and LNCaP (panel D) cells were treated with CUDC‐907 for up to 24 h and then subjected to Annexin V‐FITC/PI staining and flow cytometry analyses. Mean per cent Annexin V + cells ±standard error is shown. ***indicates *P* < .001. E‐H, LNCaP cells were infected with Precision LentiORF Mcl‐1, Bcl‐xL and RFP control or NTC‐shRNA and Bim‐shRNA lentivirus for 5 h, then washed and incubated in fresh media for 48 h prior to adding 2 μg/mL blasticidin or 1 μg/mL puromycin, respectively. Whole cell lysates of the antibiotic‐resistant cells were subjected to Western blotting and probed with the anti‐Mcl‐1, anti‐Bcl‐xL or anti‐Bim antibody (panels E&G). The fold change densitometry measurements, normalized to β‐actin and then compared to no drug treatment control, are indicated. OE, overexpression. The cells were treated with CUDC‐907 for 48 h and then subjected to Annexin V/PI staining and flow cytometry analyses (panels F&H). **indicates *P* < .01, ***indicates *P* < .001 compared to no drug treatment control, while ^##^ and ^###^indicate *P* < .01 and *P* < .001, respectively, compared to CUDC‐907‐treated RFP or NTC cells

It has been well documented that inhibition of the PI3K pathway and/or HDACs leads to changes of protein levels of the Bcl‐2 family members, such as down‐regulation of anti‐apoptotic Mcl‐1 and Bcl‐xL and up‐regulation of pro‐apoptotic Bim.^20^, ^39^, ^40^, ^41^ It is conceivable that CUDC‐907 exerts its antitumour activity against prostate cancer cells by a similar mechanism. As shown in Figure 3A,B, CUDC‐907 treatment decreased Mcl‐1 levels and increased Bim levels as early as 18 hours post drug treatment in both cell lines. Decreased Bcl‐xL protein expression was also detected starting at the 6‐hour time point in 22Rv1 cells and at the 12‐hour time point in LNCaP cells (Figure 3A,B). In contrast, Bcl‐2, Bax and Bak protein levels were unchanged post 48 hour CUDC‐907 treatment in both cell lines (Figure S2). To ensure that the changes in Bcl‐xL, Bim and Mcl‐1 were not secondary to cell death, we repeated the Annexin V/PI staining and flow cytometry analyses at earlier time points and found that the increase in Annexin V positive cells, indicative of apoptosis induction, did not occur until 18 and 24 hours post‐CUDC‐907 treatment in 22Rv1 and LNCaP cells, respectively (Figure 3C,D). These data suggest that the protein level changes in Bcl‐xL, Bim and Mcl‐1 occurred prior to or at the onset of apoptosis induction.

To further determine the role of Bcl‐xL, Bim and Mcl‐1 in apoptosis induced by CUDC‐907, overexpression of Mcl‐1 or Bcl‐xL and knockdown of Bim were performed in LNCaP cells, which were confirmed by Western blot analyses (Figure 3E,G). Annexin V/PI staining and flow cytometry analysis revealed that Mcl‐1 and Bcl‐xL overexpression and Bim knockdown partially rescued LNCaP cells from CUDC‐907‐induced apoptosis (Figure 3F,H), demonstrating that these proteins play a role in CUDC‐907‐induced apoptosis. Real‐time RT‐PCR analyses revealed that CUDC‐907 treatment caused a concentration‐dependent increase of *Bim* transcripts, but had no obvious impact on *Mcl‐1* and *Bcl‐xL* mRNA levels in both cell lines (Figure S3). These results suggest that upregulation of Bim by CUDC‐907 is likely through transcriptional mechanisms, while down‐regulation of Mcl‐1 and Bcl‐xL is likely through posttranscriptional mechanisms.

### 3.4 CUDC‐907 down‐regulates DNA damage response proteins and induces DNA damage in prostate cancer cells

**FIGURE 4:**
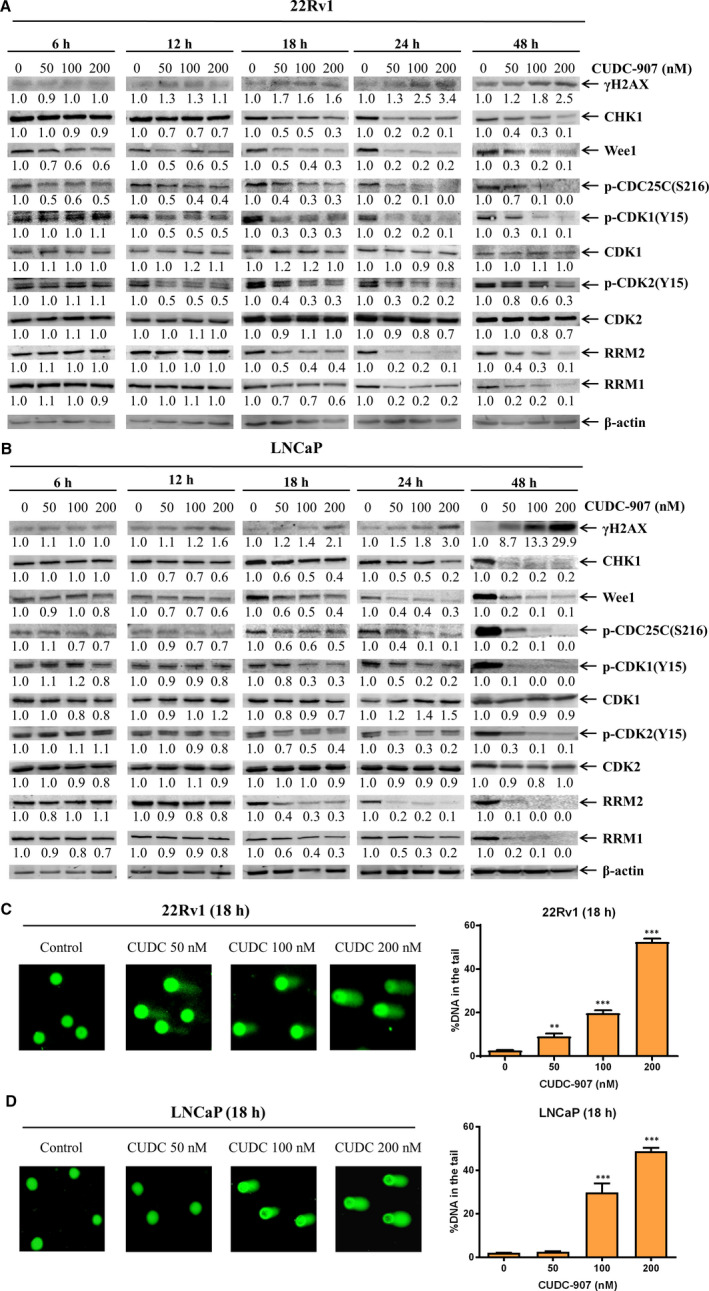
CUDC‐907 treatment down‐regulates DNA damage response proteins and induces DNA damage in prostate cancer cells. A&B, 22Rv1 (panel A) and LNCaP (panel B) cells were treated with variable concentrations of CUDC‐907 for 6‐48 h. Whole cell lysates were subjected to Western blotting and probed with the indicated antibodies. The fold changes for the densitometry measurements, normalized to β‐actin and then compared to no drug control, are indicated below the corresponding blot. C&D, 22Rv1 (panel C) and LNCaP (panel D) cells were treated with CUDC‐907 for 18 h and then subjected to alkaline comet assay analyses. Representative images are shown. Data are graphed as mean per cent DNA in the tail from 3 replicate gels ± standard error. **indicates *P* < .01, while ***indicates *P* < .001 compared to no drug treatment control

Inhibition of HDAC can down‐regulates DNA damage response (DDR) proteins, such as CHK1 and Wee1, and induces DNA damage, as we and others have previously reported.^29^, ^42^, ^43^, ^44^, ^45^ To determine if CUDC‐907 exerts its antitumour activity against prostate cancer cells via this mechanism, 22Rv1 and LNCaP cells were treated with variable concentrations of CUDC‐907 for up to 48 hours, and whole cell lysates were subjected to Western blotting. As shown in Figure 4A,B, CUDC‐907 treatment resulted in induction of γH2AX (a potential biomarker of DNA double‐strand breaks) starting at 12 hours post drug treatment, which was prior to cell death (Figure 3C,D), suggesting that CUDC‐907 treatment‐induced DNA damage in prostate cancer cells. This was accompanied by decreased CHK1, p‐CDC25C, p‐CDK1, p‐CDK2 and Wee1 (Figure 4A,B). In contrast, total CDK1 and CDK2 levels were largely unchanged throughout the 48 hours of CUDC‐907 treatment in both cell lines. In addition, RRM1 and RRM2 were also decreased in these cells starting at 18 hours post CUDC‐907 treatment (Figure 4A,B). Real‐time RT‐PCR analyses revealed that CUDC‐907 down‐regulated *CHK1*, *RRM1*, *RRM2* and *Wee1* mRNA levels as well (Figure S4). These results suggest that CUDC‐907 treatment induces DNA damage in prostate cancer cells through down‐regulation of DDR proteins via transcriptional mechanisms.

To further confirm CUDC‐907 induction of DNA damage, 22Rv1 and LNCaP cells were treated with CUDC‐907 for 18 hours and then subjected to alkaline comet assays. Consistent with the increase of γH2AX, CUDC‐907 treatment‐induced DNA strand breaks in a concentration‐dependent manner in both cell lines, reflected by the increased %DNA in the tail (Figure 4C,D). These results demonstrate that CUDC‐907 induces DNA damage potentially by transcriptionally down‐regulating CHK1, Wee1, RRM1 and RRM2 in prostate cancer cells.

### 3.5 Wee1, CHK1, RRM1 and RRM2 mediate CUDC‐907‐induced DNA damage and apoptosis

**FIGURE 5:**
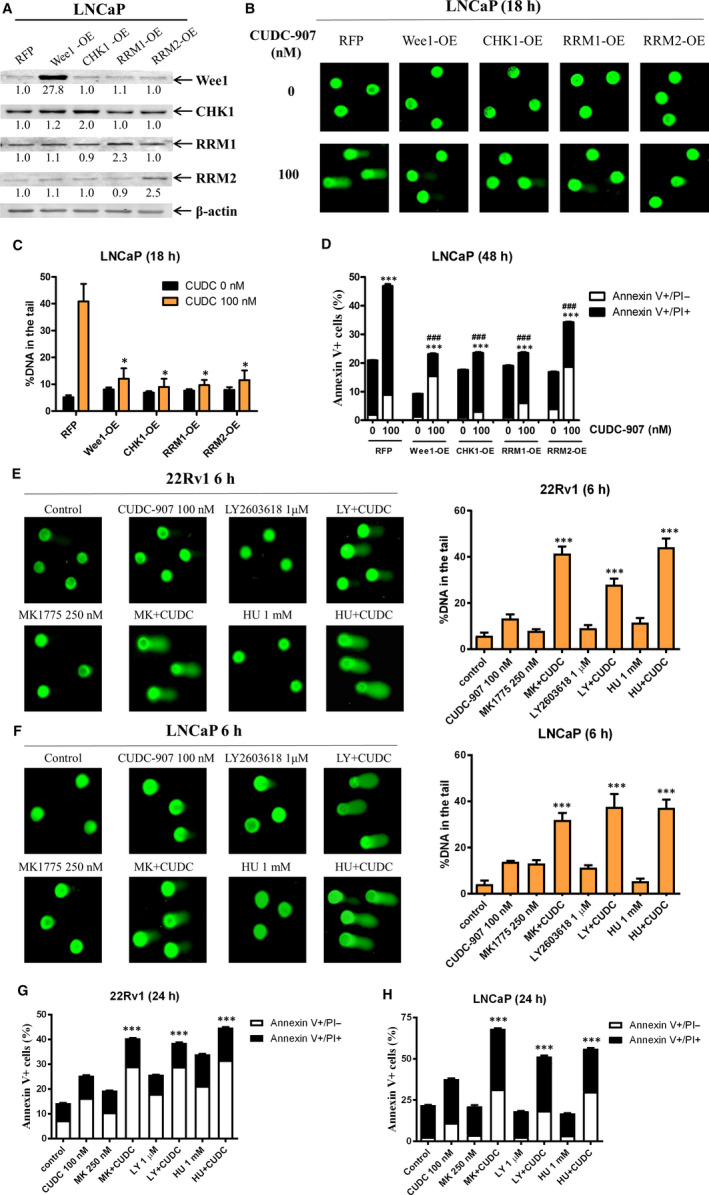
CHK1, RRM1, RRM2 and Wee1 play an important role in CUDC‐907‐induced DNA damage and apoptosis. A, LNCaP cells were infected with Precision LentiORF Wee1, CHK1, RRM1, RRM2 or RFP control lentivirus for 5 h, then washed and cultured in fresh media for 48 h prior to adding 2 μg/mL blasticidin. Whole cell lysates of the antibiotic‐resistant cells were subjected to Western blotting and probed with the indicated antibodies. The fold change densitometry measurements, normalized to β‐actin and then compared to no drug treatment control, are indicated. OE, overexpression. B&C, The antibiotic‐resistant cells were treated with 100 nmol/L CUDC‐907 for 18 h and then subjected to alkaline comet assay analyses. Representative images are shown (panel B). Data are graphed as mean per cent DNA in the tail from 3 replicate gels ± standard error (panel C). *indicates *P* < .05 compared to CUDC‐907‐treated RFP cells. D, The antibiotic‐resistant cells were treated with 100 nmol/L CUDC‐907 for 48 h and then subjected to Annexin V/PI staining and flow cytometry analysis. ***indicates *P* < .001 compared to vehicle control‐treated cells, while ^###^indicates *P* < .001 compared to CUDC‐907‐treated RFP cells. E&F, 22Rv1 (penal E) and LNCaP (panel F) were treated with CUDC‐907 alone or in combination with the Wee1 inhibitor MK‐1775, CHK1 inhibitor LY2603618 or RR inhibitor hydroxyurea for 6 h and then subjected to alkaline comet assay analyses. Representative images are shown (left panels). Data are graphed as mean per cent DNA in the tail from 3 replicate gels ± standard error (right panels). **indicates *P* < .01 and ***indicates *P* < .001 compared to the no drug treatment control and individual drug treatments. G&H, 22Rv1 (panel G) and LNCaP (panel H) were treated with CUDC‐907 alone or in combination with the Wee1 inhibitor MK‐1775, CHK1 inhibitor LY2603618 or RR inhibitor hydroxyurea for 24 h and then subjected to Annexin V/PI staining and flow cytometry analyses. ***indicates *P* < .001 compared to no drug treatment control and individual drug treatments

To provide direct evidence that CHK1, Wee1, RRM1 and RRM2 play a role in the DNA damage and apoptosis induced by CUDC‐907, Wee1, CHK1, RRM1 and RRM2 were individually overexpressed in LNCaP cells. Overexpression of Wee1, CHK1, RRM1 or RRM2, which was confirmed by Western blotting (Figure 5A), almost completely abolished CUDC‐907‐induced DNA strand breaks (Figure 5B,C) and significantly rescued the cells from CUDC‐907‐induced apoptosis (Figure 5D).

To further confirm the role CHK1, Wee1 and RR in CUDC‐907‐induced DNA damage and apoptosis, a pharmacological approach using the Wee1 inhibitor MK‐1775, CHK1 inhibitor LY2603618 and RR inhibitor hydroxyurea was utilized. As shown in Figure 5E,F, inhibition of Wee1, CHK1 or RR significantly enhanced CUDC‐907‐induced DNA strand breaks detected by alkaline comet assays. This was accompanied by significantly increased apoptosis induced by the combinations compared to individual drug treatments (Figure 5G,H). These results demonstrate that Wee1, CHK1, RRM1 and RRM2 are indeed mediators of CUDC‐907‐induced DNA damage and apoptosis in the prostate cancer cells.

### 3.6 CUDC‐907 treatment down‐regulates c‐Myc in prostate cancer cells

**FIGURE 6:**
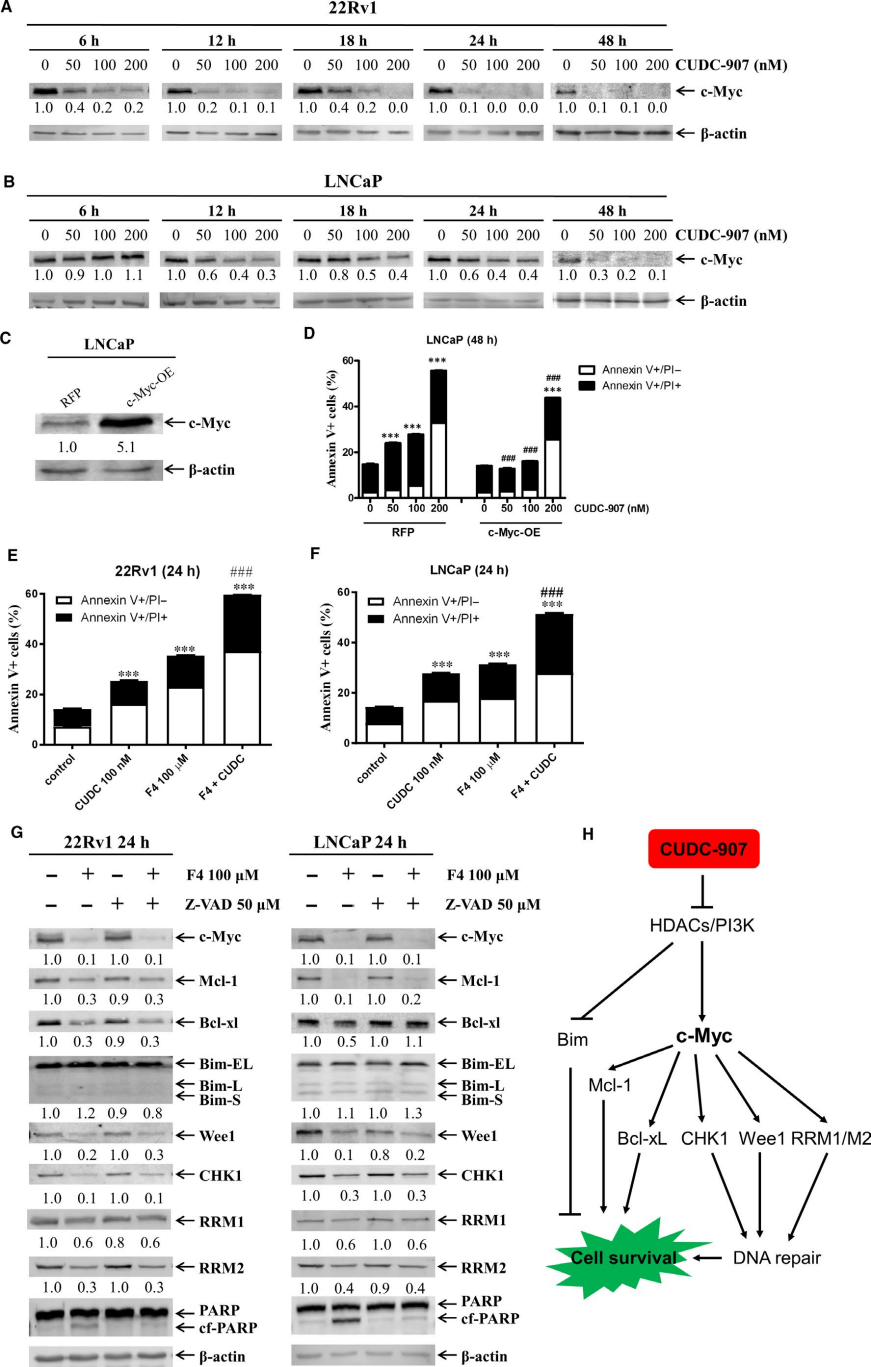
CUDC‐907 treatment down‐regulates c‐Myc in prostate cancer cells. A&B, 22Rv1 (panel A) and LNCaP (panel B) cells were treated with variable concentrations of CUDC‐907 for 6‐48 h. Whole cell lysates were subjected to Western blotting and probed with the indicated antibodies. The fold change for the densitometry measurements, normalized to β‐actin and then compared to no drug control, is indicated below the corresponding blot. C&D, LNCaP cells were infected with Precision LentiORF c‐Myc and RFP control lentivirus for 5 h, then washed and incubated for 48 h. Whole cell lysates were subjected to Western blotting and probed with the indicated antibodies (panel C). The fold change densitometry measurements, normalized to β‐actin and then compared to no drug treatment control, are indicated. OE, overexpression. The cells were treated with CUDC‐907 for 48 h and then subjected to Annexin V/PI staining and flow cytometry analysis (panel D). ***indicates *P* < .001 compared to no drug treatment control, while ^###^indicates *P* < .001 compared to CUDC‐907‐treated RFP cells. E&F, 22Rv1 (panel E) and LNCaP (panel F) were treated with CUDC‐907 alone or in combination with the c‐Myc inhibitor 10058‐F4 (F4) for 24 h and then subjected to Annexin V/PI staining and flow cytometry analyses. ***indicates *P* < .001 compared to no drug treatment control, while ^###^indicates *P* < .001 compared to individual drug treatment. G, LNCaP and 22Rv1 cells were treated with 10058‐F4 and Z‐VAD‐FMK, alone or combined, for 24 h. Whole cell lysates were subjected to Western blotting and probed with the indicated antibodies. The fold change densitometry measurements, normalized to β‐actin and then compared to no drug treatment control, are indicated. H, CUDC‐907 inhibits both HDACs and PI3K, resulting in increased expression of Bim which induces apoptosis in prostate cancer cells. Further, inhibition of HDACs and PI3K by CUDC‐907 suppresses c‐Myc expression, which likely controls the levels of Bcl‐xL, Mcl‐1, CHK1, Wee1, RRM1 and RRM2 proteins, resulting in down‐regulation of these proteins. Down‐regulation of Bcl‐xL and Mcl‐1 results in apoptosis, while down‐regulation of CHK1, Wee1, RRM1 and RRM2 results in impaired DNA repair, leading to cell death

The oncoprotein c‐Myc is frequently activated and promotes tumour development in prostate cancer,^46^, ^47^ and CUDC‐907 has been shown to down‐regulate c‐Myc in different types of cancer.^26^, ^29^, ^48^ Thus, it is possible that CUDC‐907 also exerts its antitumour activity against prostate cancer cells through down‐regulation of c‐Myc. In line with previous reports, c‐Myc protein expression was down‐regulated in prostate cancer cells as early as six hours post‐CUDC‐907 treatment in 22Rv1 and 12 hours in LNCaP, potentially through transcriptional mechanisms (Figure 6A,B and Figure S5). To determine the functional role of c‐Myc in the antitumour activity of CUDC‐907, transient overexpression of c‐Myc in LNCaP cells was performed which significantly rescued the cells from CUDC‐907‐induced apoptosis (Figure 6C,D). Conversely, pharmacologically inhibiting c‐Myc using the c‐Myc inhibitor 10058‐F4 significantly enhanced CUDC‐907‐induced apoptosis in 22Rv1 and LNCaP cell lines (Figure 6E,F). Interestingly, 10058‐F4 treatment of 22Rv1 and LNCaP cells resulted in decrease of c‐Myc (as expected), Mcl‐1, CHK1, Wee1, RRM1 and RRM2 proteins but had no obvious impact on Bim proteins in both cell lines. Further, down‐regulation of the above proteins by 10058‐F4 was independent of caspase activation (Figure 6G). Although 10058‐F4 treatment also resulted in a decrease of Bcl‐xL in both cell lines, it could be rescued by the pan‐caspase inhibitor Z‐VAD‐FMK in LNCaP cells but not in 22Rv1 cells. Taken together, these data demonstrate that c‐Myc plays an important role in CUDC‐907‐induced apoptosis in prostate cancer cells.

## 4 DISCUSSION

Resistance to AR‐targeted therapy remains a major challenge in the treatment of prostate cancer, underscoring the need for novel therapeutic approaches.^5^ Simultaneous inhibition of PI3K and HDACs has shown promising anticancer activity in solid tumours, including prostate cancer.^19^, ^20^, ^21^, ^22^ These findings provide the basis for testing the dual PI3K and HDAC inhibitor CUDC‐907 in prostate cancer. CUDC‐907 was rationally designed and synthesized by integrating HDAC inhibitory functionality (hydroxamic acid) into a core structure scaffold (morpholinopyrimidine) shared by several PI3K inhibitors. This renders CUDC‐907 dual inhibitory functionality against HDAC and PI3K.^23^ CUDC‐907 has demonstrated antitumour growth and pro‐apoptotic activity in multiple solid and haematological cancer cell lines and animal models.^23^, ^24^, ^25^, ^26^, ^29^ In addition, CUDC‐907 is being tested in Phase I and II clinical trials for the treatment of multiple myeloma, lymphoma, and advanced/relapsed solid tumours (www.clinicaltrials.gov) and showed promising clinical efficacy in relapsed/refractory lymphoma and multiple myeloma.^27^, ^28^ Here we determined, for the first time, the antitumour activity and the underlying molecular mechanisms of CUDC‐907 in prostate cancer. We show that CUDC‐907 inhibits growth and induces apoptosis of prostate cancer cells in vitro and shows promising antitumour activity in vivo in a CRPC PDX model.

Mechanistically, our results show that CUDC‐907 inhibits both PI3K and HADCs in prostate cancer cells. However, CUDC‐907 increases p‐ERK1/2 levels, which is opposite of what was observed in acute myeloid leukaemia (AML) cells.^29^ One explanation is that the PI3K/AKT and MEK/ERK pathways can negatively regulate each other's activity; inhibition of one may result in activation of the other. It has been reported that inhibition of PI3K results in up‐regulation of p‐ERK in several cancer models, including prostate cancer.^13^, ^15^, ^49^, ^50^ Activation of the MEK/ERK pathway may represent a potential resistance mechanism for CUDC‐907 treatment in prostate cancer, which could be therapeutically abolished by the ERK‐selective inhibitor SCH772984.

It has been previously reported that inhibition of the PI3K pathway and/or HDACs can result in altered protein levels of the Bcl‐2 family members.^20^, ^39^, ^40^, ^41^ The effects of CUDC‐907 on Bcl‐xL, Bim and Mcl‐1 in prostate cancer cells are not surprising because CUDC‐907 inhibits both PI3K and HDACs. Our functional studies confirm that down‐regulation of Bcl‐xL and Mcl‐1 and up‐regulation of Bim contribute to the antitumour activity of CUDC‐907 against prostate cancer cells. CUDC‐907 treatment increased *Bim* transcripts, suggesting that up‐regulation of Bim was likely through a transcriptional mechanism mediated by the HDACI moiety of CUDC‐907, as reported by Sílvia et al who indicated that HDACI is able to acetylate the Bim promoter and trigger its transcriptional activation in Mantle cell lymphoma.^51^ In contrast, CUDC‐907 treatment did not result in down‐regulation of *Mcl‐1* and *Bcl‐xL* transcripts, indicating posttranscriptional mechanisms underlying the down‐regulation of both proteins by the agent. However, the precise molecular mechanisms remain to be determined.

In addition to inducing apoptosis, CUDC‐907 treatment also resulted in DNA damage in prostate cancer cells, representing another molecular mechanism underlying apoptosis induced by this agent, as we and others have previously reported of HDACIs.^29^, ^42^, ^43^, ^44^, ^45^ Our functional studies provide direct evidence that CUDC‐907 induces DNA damage and apoptosis in prostate cancer cells at least partially through down‐regulation of CHK1, Wee1, RRM1 and RRM2. Down‐regulation of CHK1 and Wee1 would decrease the ability of prostate cancer cells to repair damaged DNA, leading to accumulation of endogenous damaged DNA, resulting in cell death if not repaired. RRM1 and RRM2 are the two subunits of RR, a key regulator of dNTP biosynthesis. Down‐regulation of RRM1 and RRM2 would result in decreased dNTP pools and impaired DNA repair.^52^ Our real‐time RT‐PCR analyses revealed that CUDC‐907 treatment significantly decreased the transcript levels for *CHK1*, *Wee1*, *RRM1* and *RRM2* in prostate cancer cells, indicating a potential transcriptional mechanism responsible for the down‐regulation of these proteins by the agent. However, the precise molecular mechanisms remain to be determined.

Transcription factor c‐Myc plays a critical role in cancer initiation and progression and is one of the most frequently deregulated oncogenes.^53^ c‐Myc is also one of the most up‐regulated genes in prostate cancer.^48^ In line with results in DLBCL and AML cells,^26^, ^29^ CUDC‐907 treatment down‐regulated c‐Myc protein and mRNA in prostate cancer cells. Transient overexpression of c‐Myc and inhibition of c‐Myc using 10058‐F4, a c‐Myc‐selective inhibitor, demonstrated a marked contribution of c‐Myc to the antitumour activity of CUDC‐907 against prostate cancer cells and suggest that c‐Myc is a potential mediator of the down‐regulation of Mcl‐1, CHK1, Wee1, RRM1 and RRM2 induced by CUDC‐907 (Figure 6H). Because the pan‐caspase inhibitor Z‐VAD‐FMK could rescue Bcl‐xL protein from 10058‐F4‐induced down‐regulation in LNCaP but not in 22Rv1 cells, c‐Myc could mediate CUDC‐907‐induced down‐regulation of the protein but in a cell type‐specific fashion. Given the critical role c‐Myc plays in cancer,^53^ down‐regulation of c‐Myc by CUDC‐907 may represent a major mechanism responsible for its antitumour activity against prostate cancer.

Reciprocal inhibition has been reported for the PI3K/AKT pathway and the AR pathway.^11^, ^54^ AR can be activated upon PI3K/AKT inhibition.^11^, ^54^ Interestingly, we did not observe any significant change in AR activity in either LNCaP or 22Rv1 cells after treatment with CUDC‐907. This might be due to CUDC‐907 inhibition of HDACs, as HDACs are known to positively regulate AR signalling.^55^

In conclusion, we demonstrate that CUDC‐907 induces apoptosis of prostate cancer cells in vitro and shows promising antitumour activity against a CRPC PDX model in vivo. Our mechanistic studies suggest that Bcl‐xL, Bim, CHK1, c‐Myc, Mcl‐1, RRM1, RRM2 and Wee1 play a role in the antitumour activity of CUDC‐907 against prostate cancer (Figure 6H). Our promising in vivo data provide a rationale for further developing CUDC‐907 for treating this disease. However, it has been previously reported that HDACIs Trichostatin A (TSA) and Suberoylanilide hydroxamic acid (SAHA) could induce epithelial‐to‐mesenchymal transition (EMT) phenotype in prostate cancer cells,^56^ therefore CUDC‐907 needs to be tested if it also possesses a similar property before its use for the treatment of prostate cancer.

## CONFLICT OF INTEREST

The authors declare that they have no competing interests.

## AUTHOR CONTRIBUTION

CH, HX, SB and JZ performed the in vitro studies. YD and YG participated in the design and coordination of the study. CH, HX, SB, JZ, HE, XL, YY, JL, GW, YZ, YD and YG participated in the data analysis and interpretation. CH, HE, GW, YD and YG helped to draft the manuscript. All authors read and approved the final manuscript.

## Supporting information

Supplementary Material

## Data Availability

The data supporting the findings of this study are available from the corresponding author upon reasonable request.
