# Supplementary material for: CUDC‐907, a novel dual PI3K and HDAC inhibitor, in prostate cancer: Antitumour activity and molecular mechanism of action
Source: J Cell Mol Med. 2020 May 27;24(13):7239–53. doi: 10.1111/jcmm.15281 (PMC7339177; doi:10.1111/jcmm.15281)
Supplement: Supplementary file 1 — Supplementary Material [file JCMM-24-7239-s001.pdf]

CUDC-907, a novel dual PI3K and HDAC inhibitor, in prostate cancer: antitumor activity and molecular mechanism of action

Cheng Hu[1#], Hongyan Xia[1#], ShanShan Bai[1,2], Jianlei Zhao[1], Holly Edwards[4,5], Xinyu Li[1], Yanrong Yang[3], Jing Lyu[1], Yang Zhan[1], Yan Dong[2]*, and Yubin Ge[4,5]*

[1]National Engineering Laboratory for AIDS Vaccine, Key Laboratory for Molecular Enzymology and Engineering, the Ministry of Education, School of Life Sciences, Jilin University, Changchun, P. R. China
[2]Department of Structural and Cellular Biology, Tulane University School of Medicine, Tulane Cancer Center, New Orleans, LA, USA
[3]School of Nursing, Jilin University, Changchun, China
[4]Department of Oncology, Wayne State University School of Medicine, Detroit, MI, USA
[5]Molecular Therapeutics Program, Barbara Ann Karmanos Cancer Institute, Wayne State University School of Medicine, Detroit, MI, USA

[#]These authors contributed equally to this work.

[*]Corresponding author

Address correspondence and reprint requests:
Yubin Ge, Ph.D.
Department of Oncology
Wayne State University School of Medicine
421 E. Canfield
Detroit, Michigan 48201, USA
Tel: (313) 578-4285
Fax: (313) 578-4287
Email: gey@karmanos.org

Or

Yan Dong, Ph.D.
1430 Tulane Avenue SL-49,
New Orleans, LA 70112, USA
Email: ydong@tulane.edu.

Table S1. Characteristics of prostate cancer cell lines

Cell lines	Sources	Prostate cancer types	AR status
LNCaP	Lymph node metastasis	Primary	AR-FL
C4-2	Lymph node metastasis derived from LNCaP in castrated mice	Castration-resistant	AR-FL
C4-2B	Bone metastasis derived from LNCaP in castrated mice	Castration-resistant	AR-FL
LAPC4	Derived from xenograft established from a lymph node metastasis	Primary	AR-FL
LNCaP 95	Derived from LNCaP in medium with charcoal-stripped fetal bovine serum	Castration-resistant	AR-FL and AR-V7
22Rv1	Derived from xenograft Line CWR22R	Castration-resistant	AR-FL and AR-Vs
DU145	Central nervous system metastasis	Castration-resistant	AR-null
PC-3	Lumbar metastasis	Castration-resistant	AR-null

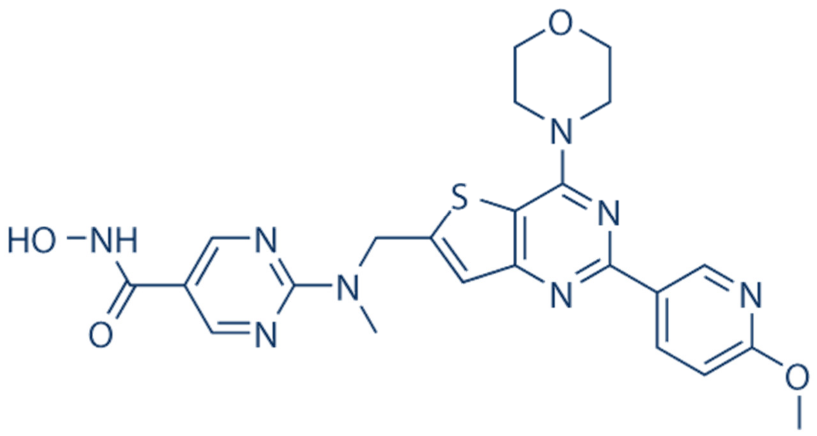

Figure S1. Chemical structure of CUDC-907.

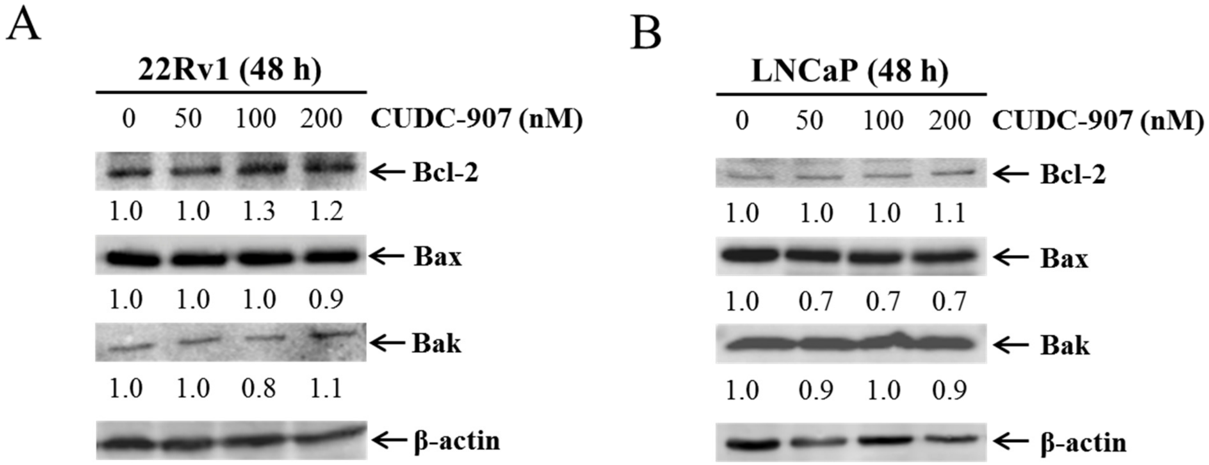

Figure S2. CUDC-907 treatment has no impact on Bcl-2, Bax, and Bak expression in prostate cancer cells. 22Rv1 (panel A) and LNCaP (panel B) cells were treated with variable concentrations of CUDC-907 for 48 hours. Whole cell lysates were subjected to Western blotting and probed with the indicated antibodies. The fold change for the densitometry measurements, normalized to β-actin and then compared to no drug control, are indicated below the corresponding blot.

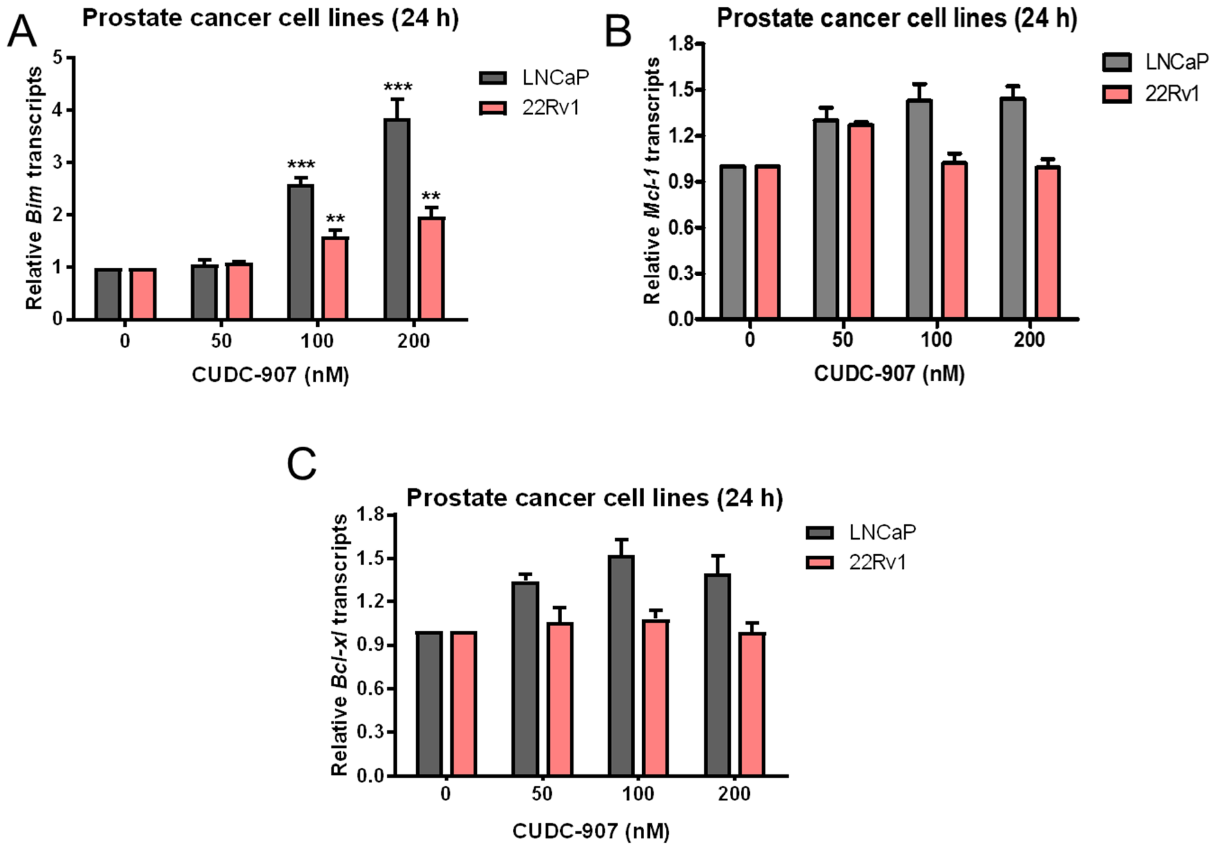

Figure S3. CUDC-907 treatment increases *Bim* transcripts, but has no negative effect on the transcript levels for *Mcl-1* and *Bcl-xL*. 22Rv1 and LNCaP cells were treated with variable concentrations of CUDC-907 for 24 hours. Total RNA was isolated and *Bim* (panel A), *Mcl-1* (panel B), and *Bcl-xL* (panel C) transcripts were measured by real-time RT-PCR. ** indicates $p<0.01$ and ***$p<0.001$ compared to no drug treatment control.

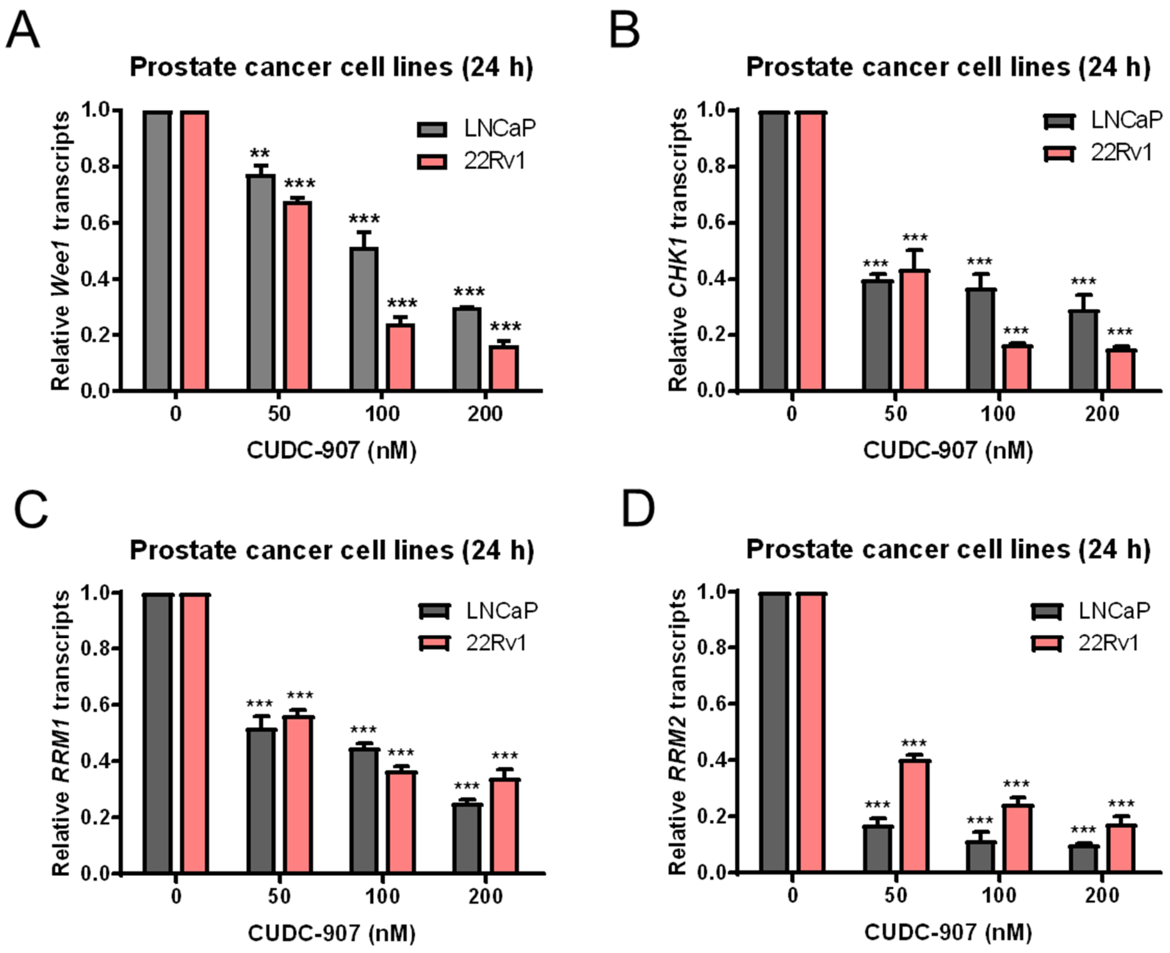

Figure S4. CUDC-907 treatment decreases *Wee1*, *CHK1*, *RRM1*, and *RRM2* transcripts in prostate cancer cells. 22Rv1 and LNCaP cells were treated with variable concentrations of CUDC-907 for 24 hours. Total RNA was isolated, and *Wee1* (panel A), *CHK1* (panel B), *RRM1* (panel C), and *RRM2* (panel D) transcripts were measured by real-time RT-PCR. *** indicates $p<0.001$ compared to no drug treatment control.

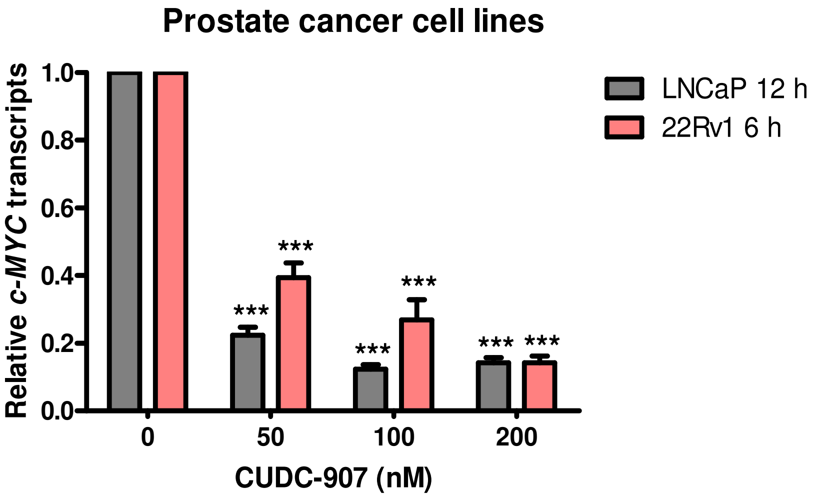

Figure S5. CUDC-907 treatment decreases *c-Myc* transcripts in prostate cancer cells. 22Rv1 and LNCaP cells were treated with variable concentrations of CUDC-907 for 6 and 12 hours, respectively. Total RNA was isolated and *c-Myc* transcripts were measured by real-time RT-PCR. *** indicates p<0.001 compared to no drug treatment control.
